# A Quantile Regression Approach Can Reveal the Effect of Fruit and Vegetable Consumption on Plasma Homocysteine Levels

**DOI:** 10.1371/journal.pone.0111619

**Published:** 2014-11-03

**Authors:** Eliseu Verly-Jr, Josiane Steluti, Regina Mara Fisberg, Dirce Maria Lobo Marchioni

**Affiliations:** 1 Department of Epidemiology, Institute of Social Medicine, Rio de Janeiro State University, Rio de Janeiro, Brazil; 2 Department of Nutrition, School of Public Health, Sao Paulo University, São Paulo, Brazil; Temple University School of Medicine, United States of America

## Abstract

**Introduction:**

A reduction in homocysteine concentration due to the use of supplemental folic acid is well recognized, although evidence of the same effect for natural folate sources, such as fruits and vegetables (FV), is lacking. The traditional statistical analysis approaches do not provide further information. As an alternative, quantile regression allows for the exploration of the effects of covariates through percentiles of the conditional distribution of the dependent variable.

**Objective:**

To investigate how the associations of FV intake with plasma total homocysteine (tHcy) differ through percentiles in the distribution using quantile regression.

**Materials and Methods:**

A cross-sectional population-based survey was conducted among 499 residents of Sao Paulo City, Brazil. The participants provided food intake and fasting blood samples. Fruit and vegetable intake was predicted by adjusting for day-to-day variation using a proper measurement error model. We performed a quantile regression to verify the association between tHcy and the predicted FV intake. The predicted values of tHcy for each percentile model were calculated considering an increase of 200 g in the FV intake for each percentile.

**Results:**

The results showed that tHcy was inversely associated with FV intake when assessed by linear regression whereas, the association was different when using quantile regression. The relationship with FV consumption was inverse and significant for almost all percentiles of tHcy. The coefficients increased as the percentile of tHcy increased. A simulated increase of 200 g in the FV intake could decrease the tHcy levels in the overall percentiles, but the higher percentiles of tHcy benefited more.

**Conclusions:**

This study confirms that the effect of FV intake on lowering the tHcy levels is dependent on the level of tHcy using an innovative statistical approach. From a public health point of view, encouraging people to increase FV intake would benefit people with high levels of tHcy.

## Introduction

The association between high concentrations of homocysteine and cardiovascular morbidity and mortality is widely recognized [1], [2]. Hyperhomocysteinemia can result from many factors: genetic (polymorphism), physiological (age and sex), lifestyle (smoking, drinking alcohol, coffee), drugs and dietary intake [3]. Dietary predictors of plasma total homocysteine levels (tHcy) include folate, B2, B6 and B12 [4], [5], [6], [7], [8].

While reliable evidence exists regarding the reduction in homocysteine concentration with supplemental folic acid, consistent evidence of the same effect for natural folate sources, such as fruit and vegetables (FV), is lacking [9], [10], [11], [12]. To date, most studies have described the overall change in the mean concentration of tHcy due to intervention, but one, by Ward et al. [13] described the effect according to tertiles of baseline plasma homocysteine concentration. In this study,, no significant response was observed In the lower tertile (mean baseline homocysteine 7.07 µmol/l), suggesting that there is a minimal level of plasma tHcy below which folic acid has no further lowering effect. In this sense, trials that found a significant effect of FV intake on Hcy have, in general, enrolled people with mean Hcy levels higher than 11 µmol/L [13], [14], [15], [16], whereas a non-significant effect was observed in studies where Hcy means were lower at baseline [9], [11], [12], [17].

The traditional statistical analysis approaches to investigate the effect of an exposure on an outcome, such as ordinary least square regression, is to compare means, and other parts of the outcome distribution do not provide further information. As an alternative, quantile regression allows for an exploration of the effects of covariates through percentiles of the conditional distribution of the dependent variable, in this case, tHcy. Thus, we aim to investigate how the associations of FV intake with tHcy differ across percentiles, i.e., from lower to higher percentiles, in the distribution of this outcome using quantile regression.

## Materials and Methods

### Study population

For the present analysis, a subsample of the population-based survey “Healthy Survey- Sao Paulo” (HS-SP), a cross-sectional study of health and living conditions among a representative sample of individuals living in São Paulo, southeastern Brazil, in 2008, was used. It was defined by eight study domains by age groups and gender. Two-stage cluster sampling of census tracts and households was performed. Further information about the sampling details has been presented in a previously published paper [18]. The HS-SP dataset contained a total of 3271 individuals aged less than 1 year and older. Of those, 2086 women and men were aged 20 years and older. For the present study, we invited all individuals older than 20 years from the HS-SP sample to answer one 24-hour recall (24HR), food frequency questionnaire (FFQ) and to have a blood sample collected. Of these, 499 individuals completed the dietary measurement and donated a blood sample for biochemical analysis.

### Data collection and processing

Information on food intake, demographics, and socioeconomic variables were obtained using structured questionnaires during household interviews. A multiple-pass 24 h recall (24HR) was administered in the household by trained interviewers. This method differs from the traditional 24HR because the interviewer uses three distinct steps to collect information about a participant’s food intake [19]. The sampling days for all participants covered all days of the week. Foods reported in each 24HR were critically reviewed to identify any failures in reporting related to the descriptions of the food consumed or to food preparation techniques, including their apportioning and quantification. The dietetic data were analyzed using Nutrition Data System for Research software version 2007 (Nutrition Coordinating Center, University of Minnesota, Minneapolis, MN, USA). In addition, a food frequency questionnaire (FFQ) was collected aiming for use to as covariate in a statistical model to correct estimates by day-today variation in dietary intake [20].

Blood samples were obtained by venipuncture after a 12 h overnight fast and immediately centrifuged, aliquoted and frozen until analysis in a freezer at −80°C. Serum folate and vitamin B12 were assayed using the Elecsys 2010 Rack Version (Roche, Switzerland) automatic electrochemiluminescence immunoassay system with Folate II and Vitamin B12 commercial kits (Elecsys and cobas analyzers, Roche Diagnostics) [21], [22]. Serum vitamin B6 levels were analyzed by an ImmunDiagnostik AG HPLC-Analytik system using high performance liquid chromatography (HPLC) with fluorometric detection [23]. Plasma total homocysteine (tHcy) levels were measured by the Immulite 2000 (Siemens, Germany) chemiluminescence Immunoassay system [24]. DNA was isolated from peripherical leukocytes and the genotypes for C677T and A1298C were determined using an allele-specific polymerase chain reaction [25], [26].

### Statistical analysis

Measurement error in dietary intake, including day-to-day variation, leads to the attenuation of regression coefficients and the loss of statistical power needed to detect associations [25]. A regression calibration as described in Kipnis et al. [20] was performed to adjust the reported intake for the within-person variation and predict usual dietary intake given the same set of covariates included in the tHcy-FV intake model. To enable this model to estimate the within-person variance, a second 24HR was administered in a random subsample (48%). The predicted usual dietary intake was then used as an explanatory variable in the tHcy-FV intake model to correct the regression coefficients. To improve the precision of the regression coefficients we included the frequency of FV consumption from the FFQ as a covariate [20].

We additionally performed a multiple linear regression using the Ordinary Least Squares (OLS) estimator to provide a basis for comparison with the quantile regression. In this model, tHcy was the dependent variable and the predicted FV intake was the independent variable, adjusted for age, sex, serum vitamin B6 and B12, predicted energy and folic acid intake, genetic variants (MTHFR C677T and MTHFR A1298C), smoking, ethylism, household *per capita* income, and an interaction between age and predicted FV intake. Further, we verified the association between tHcy and predicted FV intake using quantile regression. The set of variables was the same as those used in the OLS model described above. The coefficients were estimated for each 5 percentile from 5th to 95th, i.e., 5th, 10th, 15th,…, 90th, 95th of the dependent variable. The 95% confidence intervals were derived from standard errors generated from 200 bootstrap replications. The coefficients for each percentile were plotted and were considered to be statistically significant if their 95% CI did not cross the abscissa axis. A horizontal dashed line in the graphs indicates the OLS coefficient, and the shaded area represents its 95% CI. Quantile regression was performed in the SAS version 9.3 (SAS Corp, Cary, NC). Additionally, we plotted a graph with the predicted distribution of plasma tHcy that would be reached if individuals increased their usual FV intake by 200 g. This value is not a cut-off or a recommended amount for intake, but was used as an example of how a feasible increase in intake might affect plasma tHcy. We depart from the premise (based on our results) that increasing FV intake would reduce tHcy in different levels, according to the baseline level of tHcy. In this sense, the value showed in this figure is how a hypothetical increase in the mean FV intake in the population (e.g., increasing 200 g) would affect the percentiles of tHcy.

The study protocol was reviewed and approved by the Ethics Committee at the School of Public Health, University of Sao Paulo (Approval Number: #2001). All participants were enrolled in the study after providing free and written informed consent forms signed by themselves or by their guardians, when younger than 18 years.

## Results

A total of 499 participants are including in the study sample, and women accounted for 63% of the population. The average age was 55 (SD = 17) years, and 44% reported drinking alcohol. Nutritional status based on body mass index classification showed that 34% were overweight and 24% were obese. The mean tHcy level was 10.43 (SD = 4.35) µmol/L in the total population. For the MTHFR gene, 48% of participants had a 677 C→T mutation, 41% were CT (heterozygous) and 10% were TT (homozygous), whereas 56% presented with a 1298 A→C mutation, 36% were AC (heterozygous) and 6% were CC (homozygous).

Table 1 shows the overall means of the sample characteristics, such as diet (energy intake, FV intake, folate, methionine, betaine and choline) and other biochemical measurements (hcy, folate, B6 and B12), according to the deciles of tHcy. We can see that, as expected, the higher the tHcy, the higher the serum folate and vitamin B6; and the opposite happens to vitamin B12. However, mean dietary intake of FV, folic acid, food folate and DFE keep relatively stable through deciles of tHcy, which apparently means no or small association with tHcy.

**Table 1:** Overall means of the sample characteristics according deciles of tHcy.

			Parameters according deciles of tHcy									
	Mean	SD	1^st^	2^nd^	3^rd^	4^th^	5^th^	6^th^	7^th^	8^th^	9^th^	10^th^
***Biochemical measurements***												
Homocysteine (umol/L)	10.4	4.4	5.1	6.6	7.6	8.4	9.1	10.1	11.0	12.6	14.4	20.2
Folate (ng/mL)	10.3	4.2	11.1	11.7	10.3	11.4	9.8	10.4	10.5	9.3	9.5	9.1
Vitamin B6 (nmol/L)	60.0	24.1	47.4	53.8	54.3	54.9	58.9	62.2	64.1	64.7	64.8	75.8
Vitamin B12 (pg/mL)	307.6	198.0	317.3	347.5	352.8	362.5	347.9	278.9	323.1	277.3	238.5	222.5
***Diet***												
Energy intake (kcal)	1709.9	816.4	1831.1	1864.1	1685.4	1692.9	1748.0	1819.0	1652.1	1644.8	1619.7	1523.8
FV intake (g)	210.3	230.3	193.2	225.0	218.2	195.9	220.7	210.8	217.8	217.8	177.6	219.6
Folic Acid (mcg)	199.9	129.4	216.7	209.0	197.5	202.0	200.5	192.0	193.1	203.8	203.8	178.8
Food folate (mcg)	192.7	137.1	195.2	194.8	172.2	180.5	209.5	245.3	172.2	184.7	193.9	181.0
DFE^1^ (mcg)	533.3	286.0	564.4	550.9	508.7	524.9	551.5	572.4	501.2	532.1	541.4	485.8
Methionine (mg)	1.7	1.2	1.8	1.7	1.7	1.7	1.7	2.0	1.6	1.8	1.6	1.4
Betaine (mg)	124.1	104.5	131.7	159.7	127.2	108.7	113.6	132.6	127.4	128.2	103.7	101.9
Choline (mg)	265.4	189.2	291.6	262.5	249.2	261.0	278.2	348.7	235.2	252.2	246.3	231.6

1 DFE: Dietary folate equivalents.

**Figure 1:**
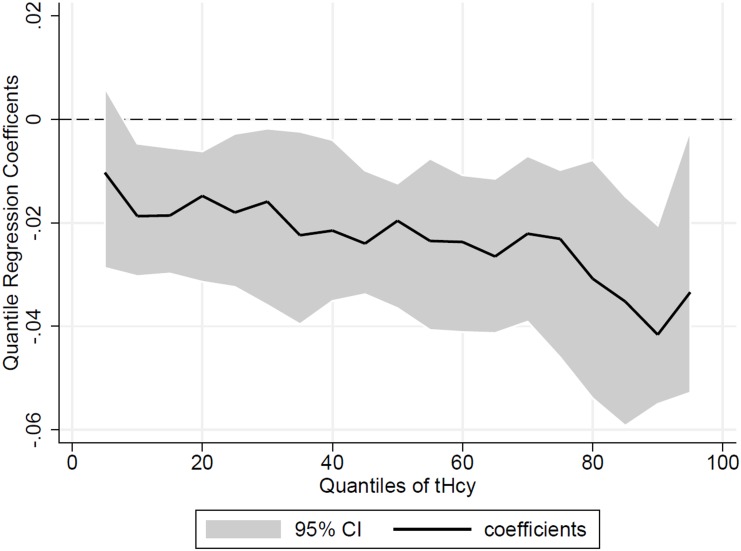
Quantile regression coefficients for fruit and vegetable intake on percentiles of plasma total homocysteine. The solid line indicates the quantile regression coefficients for FV intake on percentiles of plasma total homocysteine. Shaded area represents the 95% confidence interval for the quantile regression coefficients with their 95% CI for the relationship of tHcy and FV intake.

Figure 1 shows the quantile regression coefficients (solid line) with their 95% CI for the relationship between tHcy and FV intake. The levels of tHcy were inversely associated with FV intake when analyzed with linear regression, whereas the association occurred in a different way when quantile regression was performed. The relationship with FV consumption was inverse and significant for almost all of percentiles of plasma tHcy concentration. The coefficients were higher as the percentile of tHcy increased. For example, the quantile regression coefficient at the 85th percentile was three times the coefficient in the 20th percentile (−0.035 and −0.01 respectively). It is noticeable that in the linear regression, the estimated coefficient for this relationship was −0.025 95% CI (−0.011; −0.039) for the entire population regardless of the individual plasma tHcy concentration values.

**Figure 2:**
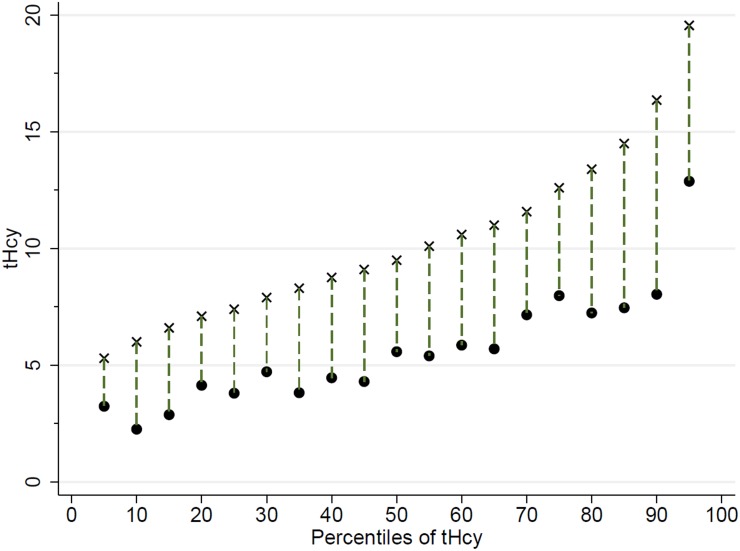
Predicted values of plasma total homocysteine for each percentile model. The prediction was based on the corresponding quantile regression model considering an increase of 200 g in the fruit and vegetable intake for each percentile. The symbols X are the baseline values of tHcy for each percentile of tHcy, and the circles are the predicted reduction for each percentile of tHcy.

Figure 2 shows the distribution of observed plasma tHcy (marked as “x”) and the predicted plasma tHcy (black circle) for a simulated increase of 200 g in FV intake. In spite of the overall decrease in the tHcy, those in the higher percentiles of tHcy would benefit more, as seen by the different lengths of the dashed vertical line in each percentile of tHcy.

## Discussion

Using a quantile regression approach, the effect of FV intake on plasma tHcy was found not to be equal throughout the distribution of tHcy. In the classical OLS approach to test this association, tHcy is expected to change as a linear function of FV intake. This means that the predicted mean and all percentiles of plasma tHcy would experience a shifting of the distribution in one direction, i.e., a change by the same coefficient. On the contrary, different coefficients were found throughout the distribution, which implies that plasma tHcy reduction by increasing FV intake will be greater in the highest percentiles.

The metabolism of homocysteine involves pathways that maintain an internal balance between homocysteine production, *i.e.,* transmethylation, and the removal process, *i.e.,* remethylation and transsulfuration, and depends on several cofactors such as methionine, B vitamins, including folate, B12, B6 and B2, betaine, and choline [27], [28]. The prevalence of inadequate folate intake has dropped since the adoption of the mandatory fortification of flour with folic acid in Brazil [29], but, at the same time, the load of methionine in this population is supposed to be high, due to the high intake of meat [30] and beans [31]. In this situation, homocysteinemia may arise from impaired methionine metabolism, and, consequently, more folate, B12 and betaine are needed to remethylation Hcy back to methionine [3].

Furthermore, an expressive number of variations of the genes regulating the enzymes that are involved in this metabolism have been identified, specifically, mutations of the MTHFR gene [32]. Individuals with the C677T mutation of the MTHFR gene, the TT genotype, usually have a higher level of tHcy than those with the CC genotype [33], but it depends on the folate [34] and B2 intake [35]. In our study, conducted in a healthy population, the intake of other B vitamins and mutations in the MTHFR gene were taken into account, so it is not probable that the deficiency of B vitamins or impaired enzyme production could explain the results.

Considering the transmethylation demand due to the methionine load, and the high intake of folic acid from mandatory fortification and adequate status of other B vitamins [29], [30], [31], the possible mechanism underlying the reduction effects of FV intake below the 75^th^ percentile could be that the homocysteine metabolism is working well, but, above this level, folate acts indirectly to lower homocysteine levels and insures optimal functioning of the remethylation pathway. However, it is known that high oral doses of folic acid can bypass the normal folate absorption mechanism [36]. In contrast to natural food folates, folic acid, *i.e.*, the fully oxidized and synthetic form of the vitamin folate used in fortified foods and supplements, need to be converted to the reduced physiological folates that are utilized by the cells [37]. Additionally, in populations with access to food fortified with folic acid, the prevalence of inadequate intake of this vitamin decreased, but the main sources of folate intake shifted from natural sources, *e.g*. vegetables, fruit and beans to the fortified foods that provide the synthetic form of folate [6], [38]. In this sense, an extra supply of folate from FV intake would benefit the remethylation pathway when the individuals present with high tHcy. Clearly, more controlled studies are needed to investigate the differences between the role of natural food folate and folic acid in the remethylation pathway, and their association them with a decrease in tHcy.

In this study we performed a quantile regression, an approach that can be particularly suitable for epidemiologists, as scientific interest often focuses on persons who have high or low – rather than average – degrees of response. It is particularly important when assessing a risk factor or biomarker that does not have a linear relationship with disease or mortality. From a public health and nutrition policy perspective, particular parts of the outcome distribution are likely to be of more interest than others, e.g., instead of focusing on the mean of the dependent variable, investigators could explore characteristics (individual, social, environmental) that are associated with this variable, which are of concern once they are linked to chronic diseases. These associations are subject to exploration even when the mean of the dependent variable is not significantly associated with a set of covariates [39], [40].

Our results should to be interpreted with some considerations. Although the coefficients have been corrected by regression calibration, this adjustment may have not been enough to remove all of the effect of the within-person variance. It is recognized that within-person variance in the dietary intake potentially attenuates regression coefficients, resulting in the underestimation of the identified coefficients. Additionally, statistical power to detect association is expected to be only partially restored after this correction. However, it is clear that this method results in a better estimate than that using either one or the average of a few collection days of 24 hr for each person in the study [20]. Conversely, we included all of the important variables that may alter plasma Hcy, such as folate and other relevant nutrients (*e.g.,* B12, B6, niacin, riboflavin, betaine and choline), genetics variations, sex, ethnicity/race, and age, in this model, suggesting the potential control of almost all factors involved in Hcy metabolism. Moreover, that the fact that people with higher intakes of FV usually are “heath conscientious” and tend to adopt a healthy life style, clustering with other health related factors that may affect Hcy levels that are not accounted for in the analysis, should not be ruled out.

This study is an exploratory hypothesis-generating investigation. Therefore, further trials studies may be reasonable to test the hypothesis identified and to elucidate the biological effect. However, this is a particular issue for trials that aim to show the association between FV intake and tHcy concentration. Many trials that fail to find a negative association between tHcy and dietary factors will not be considered ineffective if they are able to demonstrate the association in the highest values of tHcy, which are associated with cardiovascular outcomes.

The health benefits of eating fruit and vegetables are well established in the scientific society. These foods are associated with a reduced risk of coronary heart disease, several types of cancer, and some other chronic diseases [41]. Moreover, it has been suggested that a high homocysteine level is an independent predictor of cardiovascular disease [1]. In summary, we demonstrate the use of an innovative statistical approach to show that effect of FV intake on reducing tHcy is dependent on the level of plasma tHcy. From a public health point of view, encouraging people to increase FV intake would benefit people with high levels of tHcy.
